# Plasma Vitamin C and Risk of Late Graft Failure in Kidney Transplant Recipients: Results of the TransplantLines Biobank and Cohort Study

**DOI:** 10.3390/antiox10050631

**Published:** 2021-04-21

**Authors:** Camilo G. Sotomayor, Nicolas I. Bustos, Manuela Yepes-Calderon, Diego Arauna, Martin H. de Borst, Stefan P. Berger, Ramón Rodrigo, Robin P. F. Dullaart, Gerjan J. Navis, Stephan J. L. Bakker

**Affiliations:** 1 Division of Nephrology, Department of Internal Medicine, University Medical Center Groningen, University of Groningen, 9700 RB Groningen, The Netherlands; manueyepes@gmail.com (M.Y.-C.); m.h.de.borst@umcg.nl (M.H.d.B.); s.p.berger@umcg.nl (S.P.B.); g.j.navis@umcg.nl (G.J.N.); s.j.l.bakker@umcg.nl (S.J.L.B.); 2 Faculty of Medicine, University of Chile, 8330033 Santiago, Chile; nicolasbustos1995@gmail.com (N.I.B.); rrodrigo@med.uchile.cl (R.R.); 3 Department of Clinical Biochemistry and Immunohematology, Faculty of Health Sciences, Universidad de Talca, 3460000 Talca, Chile; darauna@utalca.cl; 4 Department of Endocrinology, University Medical Center Groningen, University of Groningen, 9700 RB Groningen, The Netherlands; dull.fam@12move.nl

**Keywords:** kidney transplantation, graft failure, vitamin C, oxidative stress, high-density lipoprotein, triglycerides

## Abstract

Recent studies have shown that depletion of vitamin C is frequent in outpatient kidney transplant recipients (KTR) and that vitamin C is inversely associated with risk of death. Whether plasma vitamin C is associated with death-censored kidney graft failure remains unknown. We investigated KTR who participated in the TransplantLines Insulin Resistance and Inflammation Biobank and Cohort Study. The primary outcome was graft failure (restart of dialysis or re-transplantation). Overall and stratified (*p*_interaction_ < 0.1) multivariable-adjusted Cox regression analyses are presented here. Among 598 KTR (age 51 ± 12 years-old; 55% males), baseline median (IQR) plasma vitamin C was 44.0 (31.0–55.3) µmol/L. Through a median follow-up of 9.5 (IQR, 6.3‒10.2) years, 75 KTR developed graft failure (34, 26, and 15 events over increasing tertiles of vitamin C, log-rank *p* < 0.001). Plasma vitamin C was inversely associated with risk of graft failure (HR per 1–SD increment, 0.69; 95% CI 0.54–0.89; *p* = 0.004), particularly among KTR with triglycerides ≥1.9 mmol/L (HR 0.46; 95% CI 0.30–0.70; *p* < 0.001; *p*_interaction_ = 0.01) and among KTR with HDL cholesterol ≥0.91 mmol/L (HR 0.56; 95% CI 0.38–0.84; *p* = 0.01; *p*_interaction_ = 0.04). These findings remained materially unchanged in multivariable-adjusted analyses (donor, recipient, and transplant characteristics, including estimated glomerular filtration rate and proteinuria), were consistent in categorical analyses according to tertiles of plasma vitamin C, and robust after exclusion of outliers. Plasma vitamin C in outpatient KTR is inversely associated with risk of late graft failure. Whether plasma vitamin C‒targeted therapeutic strategies represent novel opportunities to ease important burden of graft failure necessitates further studies.

## 1. Introduction

Kidney transplantation is the optimal therapy for patients with end-stage kidney disease, in terms of survival, life quality, and cost effectiveness [1,2]. Despite this success, the frequent occurrence of graft failure necessitating return to dialysis or re-transplantation remains an important medical problem that represents a constant threat for kidney transplant recipients (KTR) [3,4]. This is underscored by studies reporting that KTR regard return to dialysis as a greater threat than death [5]. Graft failure represents a major indication for dialysis or re-transplantation, in some countries with a greater contribution than any primary kidney disease, and one of the most frequent indications to start dialysis treatment in the United States [6]. Occurrence of graft failure implicates a huge socio-economic impact due to the higher costs for dialysis [7,8], decreased quality of life [9] and increased mortality risk [10]. Furthermore, taking into account, the scarcity of donor organs, prevention of re-transplantation by improving graft survival stands as an issue of paramount importance as it may translate into relief of existing organ shortage [11], underscoring a great need for identifying potentially modifiable risk factors to decrease the burden of late graft failure.

Graft failure is multifactorial, including cardiovascular disease, immunological complications, infection, and malignancy [12,13]. Oxidative stress and persistent low-grade inflammation have been associated with long-term risk of kidney graft failure [14,15]. In fact, although kidney transplantation aims to recover kidney function, oxidative stress, and chronic low-grade inflammatory status persist in outpatient KTR, partly due to maintenance immunosuppressive therapy and chronic uremic state [16,17,18,19,20,21,22]. In this setting, oxidative stress is an important contributor to cellular damage and systemic inflammation; therefore, it is believed to have a role in the underlying pathophysiological mechanisms leading to long-term graft failure [15,16,17,18,19,20,21,22,23].

Vitamin C is a recognized antioxidant and anti-inflammatory compound that attenuates oxidative stress [24], with solid pathophysiological rationale for its use in patients on renal replacement therapy and a good safety profile [25,26]. It inhibits lipid peroxidation in high-density lipoprotein (HDL) cholesterol [27,28,29]. Our group recently reported that HDL efflux capacity is associated with increased risk of graft failure in KTR, likely through an impact on progressive intra-graft vasculature atherosclerosis [30,31]. We also recently found that vitamin C depletion is prevalent in KTR and associated with increased risk of all cause-mortality [32]. However, whether vitamin C is associated with risk of graft failure and potential pathophysiological pathways involving circulating lipids associated with atherosclerosis have not yet been investigated.

Since assessment and management of vitamin C status may be widely available and inexpensive for follow-up of outpatient KTR, with foreseeable impact in clinical practice, in the present study, we aimed to investigate the association of plasma vitamin C concentrations with risk of graft failure in stable outpatient KTR.

## 2. Materials and Methods

### 2.1. Study Design and Patients

A post hoc analysis of the TransplantLines Insulin Resistance and Inflammation Biobank and Cohort Study (Clinicaltrials.gov identifier number NCT03272854) was performed. Adult KTR (≥18 years-old) being followed at the outpatient clinic, who had a functioning graft for a year or more were asked to participate between August 2001 and July 2003. Exclusion criteria included a diagnosis of overt congestive heart failure or cancer (with the exception of squamous cell or basal cell carcinoma successfully treated by a dermatologist). Eight hundred forty-seven KTR were invited to be enrolled and 606 (72%) signed written informed consent to participate. Extensive data were collected at baseline. The outpatient follow-up was performed in harmony with the American Transplantation Society guidelines [33]. For the current study, patients for whom plasma vitamin C concentration data were not available (*n* = 8) were excluded, therefore the data of 598 KTR are presented here. The Institutional Review Board approved the present study (METc 2001/039), and it was conducted following the declarations of Helsinki and Istanbul.

### 2.2. Kidney Transplant Recipients’ Characteristics

Pertinent characteristics such as sex and age of the recipient and transplant date were retrieved from the Groningen Renal Transplant Database. This database holds comprehensive information on every kidney transplantation performed at the University Medical Center Groningen from 1968 to current date. Standard immunosuppressive treatment has been described in detail previously [34]. Personal history of smoking was obtained by means of a self-report questionnaire filled by patients at inclusion. Specifics about dietary history procurement have been described priorly [35]. Collection of information regarding use of supplements containing vitamin C was performed in a systematic manner for all participants at baseline by means of self-report.

### 2.3. Clinical and Laboratory Measurements and Definitions

A complete clinical evaluation was performed and registered during a visit in morning hours to the outpatient clinic. For this visit, patients were requested to maintain a fasting period of 8–12 h because a blood sample was drawn, and they were also instructed to gather the day before the appointment a 24-h urine sample.

For the measurement of plasma vitamin C, after phlebotomy, the blood sample was immediately transported to the laboratory, while maintaining refrigeration. The sample was later deproteinized and deposited away from light at −20 °C until final processing. The measurement of ascorbic acid was performed by enzymatically transforming it to dehydroascorbic acid, which was subsequently derivatized to 3-(1,2-dihydroxyethyl) furo-[3,4-b] quinoxaline-1-one. Finally, reversed phase liquid chromatography with fluorescence detection was applied (excitation 355 nm, emission 425 nm) [36].

The methods used to determine plasma lipids, fasting glucose, insulin, and the percentage of glycated hemoglobin (HbA1C) have been extensively described before [30]. High-sensitivity C-reactive protein (hs-CRP) was determined by an enzyme-linked immunosorbent assay [37]. Class I and II anti-human leukocyte antigen antibodies (HLAab) were measured by ELISA (LATM20×5, One Lambda, Canoga Park, CA, USA) as reported previously [38]. Total urinary protein concentration was measured by means of the Biuret reaction (MEGA AU510; Merck Diagnostica, Darmstadt, Germany). Plasma creatinine concentration was determined using an adaptation of the Jaffé method (MEGA AU510; Merck Diagnostica, Darmstadt, Germany).

Estimated glomerular filtration rate (eGFR) was calculated by applying the Chronic Kidney Disease Epidemiology Collaboration equation [39]. The 2003 American Diabetes Association guidelines were used to define diabetes mellitus [40]. Urinary protein excretion that exceeded ≥0.5 g/24 h was considered proteinuria.

### 2.4. Endpoints

The primary endpoint for analyses was death-censored graft failure (return to dialysis or need for a re-transplantation). Patients were followed until 1 April 2012. Our outpatient clinic offers a continuous surveillance system, which ensured updated information on patient status and, when appropriate, cause of death. No patients were lost during follow-up.

### 2.5. Statistical Analyses

For data analyses, SPSS version 26.0 software (SPSS Inc., Chicago, IL, USA) and R version 3.2.3 (R Foundation for Statistical Computing, Vienna, Austria) were used. Data were summarized as mean (standard deviation; SD) for continuous variables with normal distribution, median (interquartile range; IQR) for continuous variables with skewed distribution, and numbers (percentage) in case of categorial variables. Differences in baseline characteristics among KTR divided by tertiles of plasma vitamin C were tested by one-way ANOVA, Mann–Whitney U test, and χ2 test in case of continuous variables with normal distribution, continuous variables with skewed distribution, and categorical variables, respectively. Next, to identify between which groups statistical differences were observed, ANOVA post hoc tests, Student’s *t*-tests, and Fisher’s exact tests were performed. In all analyses, a two-sided *p* < 0.05 was considered significant.

#### 2.5.1. Prospective Analyses

In prospective analyses, a log-rank test was performed to explore if the distribution of events by subgroups of KTR according to tertiles of plasma vitamin C was significantly different. To analyze whether plasma vitamin C was prospectively and independently associated with graft failure, we performed univariable and multivariable-adjusted Cox proportional hazards regression analyses. We performed a crude model (model 1) and afterwards we adjusted for age, sex, body mass index, donor age, and donor sex (model 2). To avoid overfitting by including an excessive amount of variables relative to the amount of events [41], other models were analyzed in such a way that further adjustments were additional to adjustments already performed in model 2 (thus, e.g., model 5 includes the additional factors of model 5 on top of the factors present in model 2). We performed further adjustments for lifestyle (physical activity, smoking status, alcohol consumption, fruit intake, and vegetable intake) in model 3; lipids (high-density lipoprotein cholesterol, low-density lipoprotein cholesterol, triglycerides, and statin use) in model 4; blood pressure, diabetes and glucose homeostasis (diabetes mellitus, glycated hemoglobin, insulin, and IRI-HOMA) in model 5; blood pressure (systolic blood pressure, number of antihypertensives, use of angiotensin-converting enzyme, and use of angiotensin II receptor blocker) in model 6; dialysis and transplant-related factors (dialysis vintage, transplant vintage, and acute rejection therapy) in model 7; and immunosuppression therapy, inflammation, and graft function (type of proliferation inhibitor, type of calcineurin inhibitor, cumulative dose of prednisolone, circulating anti-HLA class I antibodies, circulating anti-HLA class II antibodies, high-sensitivity C-reactive protein, estimated glomerular filtration rate, and proteinuria) in model 8. Proportionality assumptions of Cox proportional hazards regression analyses were met. Hazard ratios (HR) are reported with 95% confidence interval (CI). The HR of each model is given per 1-SD relative increment of vitamin C concentration. To illustrate the association of plasma vitamin C with risk of graft failure, data were fitted using median plasma vitamin C concentration as reference value.

#### 2.5.2. Sensitivity Analyses

In sensitivity analyses, we studied the association of plasma vitamin C with risk of graft failure without outliers. We identified plasma vitamin C outliers using Tukey’s fences [42], according to the formula: (Q1 − k (IQR), Q3 + k (IQR)), in which k is 1.5 for all outliers, Q1 is the lower quartile, and Q3 is the upper quartile.

#### 2.5.3. Interaction Analyses

In concordance with the recommendations for appropriate reporting on survival analyses [43,44], we evaluated potential interactions in the association between vitamin C and graft failure by fitting models containing both main effects and their cross-product terms, adjusted for the primary multivariable model of the overall prospective analyses (i.e., age, sex, body mass index, donor age, and donor sex; model 2) plus eGFR and proteinuria. For these analyses, *p*_interaction_ < 0.1 was considered to indicate significant interaction and subsequent performance of stratified analyses. For continuous variables, the cut-off points that were later used in the stratified analyses were established in such a way that the number of events in each subgroup was as comparable as feasible, and thus the statistical power for the assessment of the primary association under study in each subgroup was as much as possible similar. In secondary analyses, we also used clinically relevant cut-off points.

## 3. Results

### 3.1. Baseline Characteristics

Baseline characteristics of the study population are presented in Table 1. In total, 598 KTR (55% men, 51 ± 12 years-old, body mass index 26.0 ± 4.3 ± kg/m^2^) were included in the analyses. Mean eGFR was 47 ± 16 mL/min/1.73 m^2^. Median (IQR) plasma vitamin C was 44.0 (31.0–55.3) µmol/L. Across tertiles of plasma vitamin C distribution, we observed significant differences regarding sex (*p* < 0.001), waist circumference (*p* < 0.001), eGFR (*p* = 0.03), and proteinuria (*p* = 0.001). As for circulatory status, both systolic and diastolic blood pressure were significantly different (*p* = 0.001 and *p* = 0.003, respectively). Patients also referred significantly different fruit consumption (*p* < 0.001). Glycated hemoglobin (*p* = 0.003), insulin (*p* = 0.01), and IR-HOMA (*p* = 0.01) differed significantly among tertiles as well as hs-CRP (*p* = 0.02). Regarding lipid profile, significant differences were found among HDL cholesterol and triglycerides concentration (*p* < 0.001 and *p* = 0.01, respectively). Median time after transplantation (*p* < 0.001), use of calcineurin inhibitors (*p* = 0.01), and cumulative prednisolone dose (*p* < 0.001) also differed significantly among tertiles (Table 1).

### 3.2. Prospective Analyses

**Figure 1:**
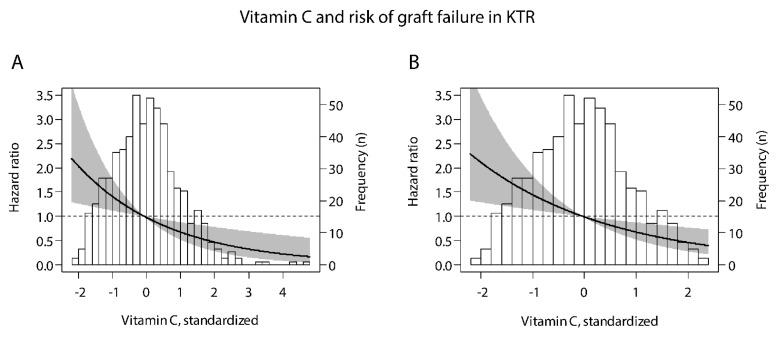
Associations of plasma vitamin C with risk of graft failure in kidney transplant recipients, within the (**A**) whole study population and (**B**) after exclusion of outliers of the distribution of plasma vitamin C (*n* = 10). To create graph B, the same analysis was performed as for graph A, but after exclusion of outliers. *X*-axis represents plasma vitamin C concentration, and *y*-axis represents the estimated hazard ratios using median plasma vitamin C (44 µmol/L) as reference value. Data were fitted by Cox proportional-hazards regression. The black line represents the hazard ratio and the gray area represents the 95% confidence interval. The histogram of plasma vitamin C is provided in the background. Patients with plasma vitamin C lower and higher than the median were, respectively, at lower and higher risk of graft failure.

During a median follow-up of 9.5 (6.3–10.2) years, 75 (13%) patients developed graft failure (34, 26, and 15 events over increasing tertiles of plasma vitamin C, log rank test *p* < 0.001). Univariate Cox proportional-hazards regression analyses of the association between tertiles of plasma vitamin C concentration and graft failure showed that patients in the highest tertile of plasma vitamin C when compared to those in the lowest tertile had a lower risk of developing graft failure (Table 2, HR 0.37; 95% CI 0.20–0.68). This association remained independent of adjustment for potential confounders, including age, sex, body mass index, donor age, and donor sex (with e.g., an HR of 0.36; 95% CI 0.19–0.67 in model 2). Further adjustment for donor, recipient, and transplant characteristics, including eGFR and proteinuria did not materially alter the association. Vitamin C, as a continuous variable, was also inversely associated with risk of graft failure (HR 0.69; 95% CI 0.54–0.89 per 1-SD relative increment, *p* = 0.004; Figure 1A). Likewise, in further multivariable-adjusted analyses, the association remained materially unchanged (Table 2).

### 3.3. Sensitivity Analyses

The prospective association of plasma vitamin C with risk of graft failure without outliers is illustrated in Figure 1B. In these analyses, we consistently found that patients with plasma vitamin C lower and higher than the median were at higher and lower risk of graft failure, respectively.

### 3.4. Interaction Analyses

**Figure 2:**
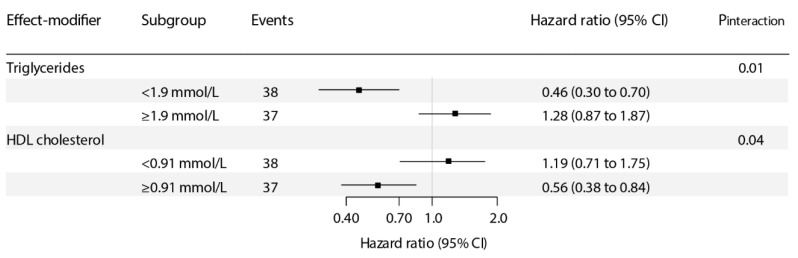
Stratified prospective analyses of the association of plasma vitamin C with risk of graft failure. *p*_interaction_ was calculated by fitting models, which contain both main effects and their cross-product term, with adjustment for age, sex, body mass index, donor age, donor sex, estimated glomerular filtration rate (eGFR), and proteinuria. Hazard ratios (95% CI) are calculated per 1–SD increment in plasma vitamin C.

For the association of plasma vitamin C with risk of graft failure, we found signs of a significant interaction with triglycerides and HDL cholesterol (*p*_interaction_ = 0.01 and 0.04, respectively; Table S1). In stratified analyses, we found that plasma vitamin C was inversely associated with risk of graft failure particularly in KTR with triglycerides concentration of <1.9 mmol/L (HR, 0.46; 95% CI, 0.30–0.70 per 1-SD relative increment; *p* < 0.001; Figure 2) or <1.7 mmol/L (common clinical cut-off point; HR, 0.49; 95% CI, 0.30–0.79 per 1-SD relative increment; *p* < 0.003; Table S2) and HDL cholesterol concentration of ≥0.91 (HR, 0.56; 95% CI 0.38–0.84 per 1-SD relative increment; *p* = 0.01; Figure 2) and ≥1.1 mmol/L (common clinical cut-off point; HR, 0.41; 95% CI 0.25–0.68 per 1-SD relative increment; *p* = 0.001; Table S2). Similar results on interaction analyses were found over tertiles of plasma vitamin C (Table 3 and Table S2).

## 4. Discussion

Our results consistently show that outpatient KTR in the highest tertile of plasma vitamin C are at significantly lower risk of long-term kidney graft failure, independently of donor, recipient, and transplant characteristics, including eGFR and proteinuria. Likewise, in continuous analyses of plasma vitamin C, we observed a consistent inverse association with risk of graft failure. The association was robust after exclusion of outliers, and particularly strong in patients with relatively lower triglycerides and relatively higher HDL cholesterol concentration. These results suggest that plasma vitamin C is an independent risk factor for long-term graft failure in outpatient KTR, pointing towards the need for further evaluating potential underlying mechanisms linking plasma vitamin C with lower risk of graft failure, while suggesting the involvement of mechanisms pertaining to the effect of circulating lipids on risk of graft failure, particularly HDL cholesterol and triglycerides.

Vitamin C is a well-known antioxidant and anti-inflammatory compound that attenuates oxidative stress and lipid peroxidation by scavenging reactive oxygen species [24]. We have previously investigated the anti-inflammatory and anti-oxidant agent vitamin C in outpatient KTR. We first evaluated the prevalence of patients with plasma levels within the range of depletion (≤28 µmol/L or 0.5 mg/dL) in order to assess its potential association with risk of mortality [32]. We found that vitamin C depletion was common (22%) in otherwise stable outpatient KTR, which independently associated with an almost two-fold increased risk of overall mortality. Due to our interest on the involvement of chronic low-grade inflammation in adverse long-term outcomes of KTR [45], we furthermore studied the potential mediation effect of predefined inflammatory biomarkers and found that approximately one-third of the association of vitamin C with mortality risk may be explained by a composite score of inflammatory biomarkers [32], supporting the notion that a beneficial effect of vitamin C on long-term outcomes of KTR occurs, at least to a considerable extent, through decreasing the chronic low-grade inflammatory status.

In line with extensive literature arguing for beneficial effects of vitamin C treatment in cancer prevention [46], Gacitúa et al. recently underscored the sizeable relevance of plasma vitamin C as a novel potentially modifiable risk factor for premature mortality post-kidney transplantation by describing an independent inverse association with risk of death, particularly death due to malignancy [47], potentially by counteracting toxic effects of maintenance immunosuppression therapy, specifically calcineurin inhibitors, which are the most accepted risk factor for de novo malignancy post-kidney transplantation [48]. Interestingly, in the current study, we observed signs of an inverse relationship between use of calcineurin inhibitors and plasma vitamin C, which may be in line with the data of the two randomized clinical trials of vitamin C performed in KTR, reporting an inverse association between vitamin C supplementation and cyclosporine A trough levels [49,50]. It should be realized, however, that this otherwise unwanted effect of vitamin C supplementation post-kidney transplantation has not been shown to have impacted negatively actual outcomes of KTR. In fact, efforts are up until now made to decrease the toxic effect of maintenance immunosuppressive therapy. Whether vitamin C may counterbalance such effects and decrease the burden of premature mortality in KTR—as suggested by the aforementioned observational studies reporting an inverse association between plasma vitamin C and risk of death—remains unknown.

Although moderate doses of vitamin C supplementation (up to 1 g/d) are considered safe, vitamin C supplementation is not exempt of potential drawbacks such as oxalosis, of which its appearance would largely depend on initial (pre-intervention) vitamin C status [51,52]. To the best of our knowledge, previous studies (in different clinical settings) have performed randomized supplementation of fixed doses of vitamin C, despite initial vitamin C status. It should be realized that while the current study suggests a linear association between plasma vitamin C and risk of graft failure, according to our analyses on the association of vitamin C and all-cause mortality, the therapeutic potential of a pharmacological supplementation strategy in non-depleted patients may be relatively lower compared to patients with sub-physiological vitamin C status, while vitamin C deficient patients may need higher supplementation doses to reach physiological levels and make apparent the benefits from intervention. While we emphasize that commonly recommended vitamin C intake of 40 mg/d for adults can be obtained from a healthy diet, we also underscore that future investigations aiming to explore the potential of pharmacological intervention strategies could best (i) take into account initial vitamin C status, (ii) consider individualized vitamin C supplementation, (iii) monitor vitamin C status in order to adhere to reference values (28–85 µmol/L or 0.5–1.5 mg/dL) [53], and (iv) avoid high-dose vitamin C therapy, as it is known that impaired kidney function limits normal excretion of excess vitamin C, which may lead to secondary oxalosis with potential deleterious consequences, including graft failure [54].

As for potential underlying mechanisms of our main finding, and the observed effect-modification by triglycerides and HDL cholesterol, it should be realized that progressive atherosclerosis in the grafts vasa, a phenomenon called transplant vasculopathy, is an important pathogenic factor of chronic kidney transplant dysfunction, recognized as one of the main causes of graft failure passed the first year post-kidney transplantation [55,56]. High triglycerides and low HDL cholesterol are well-established cardiovascular risk factors for progressive atherosclerosis. One described beneficial effect of vitamin C is by inhibiting lipid peroxidation in HDL, thus preserving its antioxidant activity [25], as oxidation of HDL impairs cholesterol efflux capacity and its cardioprotective effect [26,27]. Although differences in HDL-cholesterol between KTR according to increasing tertiles of vitamin C, with respective mean values of 1.0, 1.1, and 1.2 mmol/L, may seem numerically marginal at first sight, it should be noted that small differences in HDL cholesterol may already be associated with relatively large differences in incidence of atherosclerosis-related disease. An example may be the combined data from the Framingham study, the Coronary Primary Prevention Trial, and Multiple Risk Factor Intervention Trial, which have previously shown that each increase in HDL cholesterol of 1 mg/dL (i.e., 0.03 mmol/L) associates with a decrease in the risk of coronary heart disease of 2% in men and 3% in women in the general population [57,58]. Our group recently reported that low levels of HDL are associated with risk of graft failure in KTR and that HDL efflux capacity was a strong predictor of this adverse outcome, likely through a beneficial impact on progressive atherosclerosis in the vasculature of the transplanted kidney [28,29]. Indeed, oxidative stress is a key mediator for endothelial dysfunction and atherosclerosis, and may be counteracted by preservation of nitric oxide (NO) [59,60,61,62,63], which is a vasodilator and anti-atherogenic molecule that maintains vascular homeostasis and modulates lipid peroxidation [64]. Vitamin C plays a role in the exogenous pathway of NO production [65]. It works as an enhancer to catalyze the conversion of acidified nitrite to NO, resulting in an increased NO pool [62,66,67], thus linking vitamin C-targeted interventions with augmentation of NO, reduction in oxidative stress, and anti-atherosclerotic effects. By scavenging reactive oxygen species, vitamin C also facilitates inhibition of lipid peroxidation of HDL, preserving, in turn, antioxidant activity of HDL [29], which is relevant for its cholesterol efflux ability and protective effect on the vasculature [27,28], contributing to prevent or reverse intragraft atherosclerosis, and thereby slowing the decline in kidney function. These findings are in agreement with the aforementioned previous report of our group [30,31]. Hence, the current study further underscores the need for evaluating the therapeutic potential of vitamin C-targeted interventional strategies to decrease the burden of long-term adverse outcomes in outpatient KTR.

We have, here, presented a prospective cohort study in a large sample of outpatient KTR, who were strictly monitored during a substantial follow-up period by frequent check-up in the outpatient clinic, allowing for complete endpoint evaluation without follow-up losses. Further strengths of the study are that our findings were robust in sensitivity analyses with exclusion of outliers. In addition, having extensively collected baseline data available allowed us to perform analyses with adjustment for multiple potential confounders. Although the prospective analyses were adjusted for several lifestyle factors, including exercise (physical activity) and consumption of healthy dietary items (i.e., fruit and vegetable intake), we did not take into account more granular data on dietary habits or calculated nutritional intake to evaluate the potential interplay of various nutrients. Due to the observational nature of the current study, it does not allow for assumptions on causality and residual confounding remains possible, due to lack of adjustment for unmeasured variables (e.g., intake or circulating levels of other micro- and macronutrients). It also is not possible to exclude that the association exists as a consequence of vitamin C being a marker of poor nutrition. Finally, the majority of the study population was Caucasian, which calls for prudence when extrapolating these results to different populations.

## 5. Conclusions

In conclusion, plasma vitamin C is inversely associated with risk of long-term graft failure in KTR, independently of donor, recipient, and transplant characteristics, including eGFR and proteinuria. We found signs of a potential pathophysiological pathway involving circulating lipids (triglycerides and HDL cholesterol). Whether timely management of plasma vitamin C levels may be a target to decrease the risk of long-term graft failure among outpatient KTR needs to be further studied.

## Figures and Tables

**Table 1:** Baseline characteristics of 598 kidney transplant recipients according to tertiles of plasma vitamin C.

	Plasma Vitamin C, Tertiles						*p*
	Tertile 1 (*n* = 197)		Tertile 2 (*n* = 202)		Tertile 3 (*n* = 199)		
	<35 µmol/L		35‒51 µmol/L		≥51 µmol/L		
Plasma vitamin C, µmol/L	23.4	(8.2)	43.5	(4.6)	66.3	(13.9)	–
**Demographics**							
Age, years, mean (SD)	52	(12)	51	(12)	52	(12)	0.70
Sex, male, *n* (%)	129	(66)	117	(58)	82	(41) †‡	<0.001
Caucasian ethnicity, *n* (%)	190	(96)	192	(95)	195	(98)	0.28
Body mass index, kg/m^2^, mean (SD)	26.4	(4.8)	26.0	(4.1)	25.6	(4.0)	0.21
Waist circumference, cms, mean (SD) ^a^	99.9	(13.8)	96.6	(13.5)	94.8	(13.4) †	<0.001
**Kidney allograft function**							
eGFR, mL/min/1.73 m^2^, mean (SD)	44	(15)	48	(17)	49	(15) †	0.03
Proteinuria ≥0.5 g/24 h, *n* (%) ^b^	70	(36)	59	(29)	37	(19) †‡	0.001
**Cardiovascular history**							
History of cardiovascular disease, *n* (%) ^c^	25	(13)	26	(13)	23	(12)	0.89
Systolic BP, mmHg, mean (SD)	158	(25)	152	(21) †	150	(22) †	0.001
Diastolic BP, mmHg, mean (SD)	92	(10)	89	(10) †	89	(9) †	0.003
Use of antihypertensives, *n* (%)	177	(90)	180	(89)	165	(83)	0.07
Use of ACE inhibitor or ARB, *n* (%)	68	(35)	133	(66)	135	(68)	0.87
Use of β-blockers, *n* (%)	124	(63)	124	(61)	120	(60)	0.86
Use of calcium-antagonists, *n* (%)	78	(40)	80	(40)	70	(35)	0.58
**Lifestyle**							
Current or former-smoker, *n* (%)	130	(66)	131	(65)	121	(61)	0.53
*Alcohol use*							0.57
None, *n* (%) ^d^	95	(48)	92	(46)	96	(48)	
1‒7 units/week, *n* (%) ^d^	72	(37)	83	(41)	66	(33)	
>7 units/week, *n* (%) ^d^	27	(14)	25	(12)	32	(16)	
Physical activity, MET-min/day, median (IQR) ^e^	197	(34–562)	286	(68–647)	212	(46–549)	0.69
Fruit consumption, servings/day, median (IQR) ^f^	1	(1–2)	2	(1–2) †	2	(1–2) †	<0.001
Vegetable consumption, tablespoons/day, median (IQR) ^f^	2	(2–3)	3	(2–3)	2	(2–3)	0.23
**Diabetes and glucose homeostasis**							
Diabetes mellitus, *n* (%)	42	(21)	38	(19)	25	(13)	0.06
HbA_1C_, %, mean (SD) ^a^	6.6	(1.1)	6.5	(1.1)	6.4	(1.0)	0.003
Insulin, µU/mL, median (IQR)	11.9	(7.9–16.8)	11.2	(8.3–17.0)	10.8	(7.7–14.8) ‡	0.01
HOMA-IR, score, median (IQR)	2.3	(1.6–3.9)	2.3	(1.7–3.8)	2.2	(1.5–3.2) ‡	0.01
**Laboratory measurements**							
hs-CRP, mg/L, median (IQR)	3.1	(1.4–7.4)	1.5	(0.5–4.1) †	1.8	(0.8–4.0) †	0.02
Total cholesterol, mmol/L, mean (SD)	5.6	(1.3)	5.7	(1.1)	5.6	(0.8)	0.40
HDL cholesterol, mmol/L, mean (SD)	1.0	(0.3)	1.1	(0.3)	1.2	(0.4) †‡	<0.001
LDL cholesterol, mmol/L, mean (SD)	3.5	(1.2)	3.6	(1.0)	3.5	(0.7)	0.30
Triglycerides, mmol/L, mean (SD)	2.3	(1.1)	2.2	(1.5)	2.0	(1.1) †	0.01
**Kidney transplant and immunosuppressive therapy**							
*Dialysis vintage*							
<1 year, *n* (%)	42	(21)	44	(22)	55	(28)†	0.04
1–5 year, *n* (%)	115	(58)	129	(64)	119	(60)†	
>5 year, *n* (%)	40	(20)	29	(14)	25	(13)†	
Time since transplantation, years, median (IQR)	4.6	(1.9–9.5)	5.9	(2.7–10.6) †	7.6	(3.8–13.9) †‡	<0.001
Donor type (living), *n* (%)	26	(13)	27	(13)	30	(15)	0.84
Use of calcineurin inhibitor, *n* (%)	168	(85)	156	(77) †	146	(73)†	0.01
Use of proliferation inhibitor, *n* (%)	140	(71)	149	(74)	152	(76)	0.23
Cumulative prednisolone, grams, median (IQR) ^b^	16.7	(8.4–31.5)	21.4	(11.5–37.9) †	14.8	(23.7–44.8) †	<0.001

Data available in ^a^ 597, ^b^ 596, ^c^ 594, ^d^ 588, ^e^ 540, and ^f^ 400 patients. † Significantly different from tertile 1; ‡ significantly different from tertile 2. ACE, angiotensin converting enzyme; ARB, angiotensin II receptor blocker; HbA_1C_, hemoglobin A1C; HOMA-IR, homeostasis model assessment of insulin resistance; hs-CRP, high-sensitivity C-reactive protein.

**Table 2:** Prospective association of plasma vitamin C with risk of graft failure.

Models	Plasma Vitamin C				
	Tertiles			Per 1-SD, Relative Increment	
	Tertile 1	Tertile 2	Tertile 3		
	Ref.	HR (95% CI)	HR (95% CI)	HR (95% CI)	*p*
Model 1	1.00	0.68 (0.41–1.13)	0.37 (0.20–0.68)	0.69 (0.54–0.89)	0.004
Model 2	1.00	0.64 (0.38–1.07)	0.36 (0.19–0.67)	0.67 (0.51–0.87)	0.002
Model 3	1.00	0.61 (0.32–1.17)	0.32 (0.15–0.70)	0.61 (0.44–0.85)	0.004
Model 4	1.00	0.64 (0.38–1.08)	0.39 (0.21–0.73)	0.68 (0.52–0.89)	0.005
Model 5	1.00	0.66 (0.39–1.10)	0.38 (0.20–0.71)	0.69 (0.53–0.91)	0.007
Model 6	1.00	0.69 (0.40–1.16)	0.42 (0.22–0.80)	0.70 (0.53–0.92)	0.011
Model 7	1.00	0.45 (0.17–1.14)	0.25 (0.08–0.78)	0.54 (0.33–0.88)	0.013
Model 8	1.00	0.70 (0.40–1.22)	0.42 (0.21–0.83)	0.72 (0.54–0.95)	0.022

Cox proportional-hazards regression analyses were performed to assess the association of plasma vitamin C concentration with risk of graft failure (number of events = 75). Associations are shown with plasma vitamin C concentration as a continuous variable and according to tertiles of the vitamin C distribution (tertile 1, *n* = 197: <35 µmol/L; tertile 2, *n* = 202: 35‒51 µmol/L; tertile 3, *n* = 199: ≥51 µmol/L). Model 1 was unadjusted. Multivariable model 2 was adjusted for age, sex, body mass index, donor age, and donor sex. Subsequently, additive adjustments were performed based on the variables already adjusted for in model 2, with additional adjustment for lifestyle (physical activity, smoking status, alcohol consumption, fruit intake, and vegetable intake) in model 3; lipids (high-density lipoprotein cholesterol, low-density lipoprotein cholesterol, triglycerides, and statin use) in model 4; blood pressure, diabetes and glucose homeostasis (diabetes mellitus, glycated hemoglobin, insulin, and IRI-HOMA) in model 5; blood pressure (systolic blood pressure, number of antihypertensives, use of angiotensin-converting enzyme, and use of angiotensin II receptor blocker) in model 6; dialysis and transplant-related factors (dialysis vintage, transplant vintage, and acute rejection therapy) in model 7; immunosuppression therapy, inflammation, and graft function (type of proliferation inhibitor, type of calcineurin inhibitor, cumulative dose of prednisolone, circulating anti-HLA class I antibodies, circulating anti-HLA class II antibodies, high-sensitivity C-reactive protein, estimated glomerular filtration rate, and proteinuria) in model 8.

**Table 3:** Stratified prospective analyses of the association of plasma vitamin C with risk of graft failure.

Models	Plasma Vitamin C		
	Tertiles		
	Tertile 1	Tertile 2	Tertile 3
	Ref.	HR (95% CI)	HR (95% CI)
**Triglycerides (*p*_interaction_ = 0.01)**			
<1.9 mmol/L (*n*_events_ = 38)	1.00	0.38 (0.18–0.79)	0.10 (0.03–0.36)
≥1.9 mmol/L (*n*_events_ = 37)	1.00	1.18 (0.49–2.56)	1.97 (0.79–4.89)
**HDL (*p*_interaction_ = 0.04)**			
<0.91 mmol/L (*n*_events_ = 37)	1.00	0.79 (0.35–1.79)	2.09 (0.84–5.17)
≥0.91 mmol/L (*n*_events_ = 38)	1.00	0.51 (0.24–1.05)	0.17 (0.05–0.51)

Cox proportional-hazards regression analyses were performed to assess the association of plasma vitamin C concentration with risk of graft failure (*n*_events_ = 75). Associations are shown with plasma vitamin C concentration as a continuous variable and according to tertiles of the vitamin C distribution (tertile 1, *n* = 197: <35 µmol/L; tertile 2, *n* = 202: 35‒51 µmol/L; tertile 3, *n* = 199: ≥51 µmol/L), with adjustment for age, sex, body mass index, donor age, donor sex, eGFR, and proteinuria.

## Data Availability

Not applicable.

## Supplementary Materials

The following are available online at https://www.mdpi.com/article/10.3390/antiox10050631/s1, Table S1: Potential effect-modifiers of the association between plasma vitamin C and risk of graft failure, Table S2: Stratified prospective analyses of the association of plasma vitamin C with risk of graft failure, using clinically relevant cut-off points.
