# Supplementary material for: Plasma Vitamin C and Risk of Late Graft Failure in Kidney Transplant Recipients: Results of the TransplantLines Biobank and Cohort Study
Source: Antioxidants (Basel). 2021 Apr 21;10(5):631. doi: 10.3390/antiox10050631 (PMC8143099; doi:10.3390/antiox10050631)
Supplement: Supplementary file 1 [file antioxidants-10-00631-s001.zip › antioxidants-1172521-supplementary.pdf]

Plasma Vitamin C and Risk of Late Graft Failure in Kidney Transplant Recipients: Results of the TransplantLines Biobank and Cohort Study

Camilo G. Sotomayor, MD, PhD[1,2]; Nicolás I. Bustos, BSc[2]; Manuela Yepes-Calderón, MD[1]; Diego Arauna, PhD[3]; Martin H. de Borst, MD, PhD[1]; Stefan P. Berger, MD, PhD[1]; Ramón Rodrigo, MSc[2]; Robin P.F. Dullaart, MD, PhD[4]; Gerjan J. Navis, MD, PhD[1]; Stephan J.L. Bakker, MD, PhD[1]; TransplantLines Investigators

[1]Division of Nephrology, Department of Internal Medicine, University Medical Center Groningen, University of Groningen, Groningen, the Netherlands; [2]Faculty of Medicine, University of Chile, Santiago, Chile; [3]Department of Clinical Biochemistry and Immunohaematology, Faculty of Health Sciences, Universidad de Talca, Talca, Chile; [4]Department of Endocrinology, University of Groningen, Groningen, the Netherlands

Correspondence: Dr. Camilo G. Sotomayor. Division of Nephrology, Department of Internal Medicine, University Medical Center Groningen, Hanzeplein 1, P.O. Box 30.001, 9713 GZ Groningen, the Netherlands. Tel:31-050-361-1564. E-mail: c.g.sotomayor.campos@umcg.nl.

Table S1. Potential effect-modifiers of the association between plasma vitamin C and risk of graft failure

Potential effect-modifiers	Plasma vitamin C, per 1-SD increment	
	**B**	***P***
Age, years	0.01	0.28
Sex (male)	0.35	0.24
eGFR, mL/min/1.73 m^2	<0.001	0.68
Proteinuria ≥0.5 g/24 h	−0.29	0.29
Systolic blood pressure, mmHg	0.002	0.69
Fruit consumption, servings/day	0.04	0.74
Vegetable consumption, tablespoons/day	−0.04	0.83
hs-CRP, mg/L	0.001	0.98
Triglycerides, mg/dL	0.28	0.01
HDL cholesterol, mg/dL	−1.11	0.04
LDL cholesterol, mg/dL	−0.16	0.34
Dialysis vintage	0.65	0.11
Time since transplantation, years	0.004	0.86
Use of calcineurin inhibitor	−0.13	0.20
Cumulative prednisolone, grams	0.002	0.82

Cox proportional-hazards regression analyses were performed with adjustment for age, sex, body mass index, donor age, donor sex, eGFR and proteinuria.

Table S2. Stratified prospective analyses of the association of plasma vitamin C with risk of graft failure, using clinically relevant cut-off points

Models	Plasma vitamin C				
	Tertiles			Per 1-SD, relative increment	
	Tertile 1	Tertile 2	Tertile 3		
	Ref.	HR (95% CI)	HR (95% CI)	HR (95% CI)	*P*
**Triglycerides (P_{int}=0.01)**					
<1.7 mmol/L (n_e=29)	1.00	0.58 (0.25—1.33)	0.16 (0.04—0.61)	0.49 (0.30—0.79)	0.003
≥1.7 mmol/L (n_e=46)	1.00	0.75 (0.37—1.53)	1.04 (0.46—2.31)	1.05 (0.73—1.51)	0.80
**HDL (P_{int}=0.04)**					
<1.1 mmol/L (n_e=52)	1.00	0.82 (0.42—1.58)	1.29 (0.60—2.80)	1.08 (0.77—1.53)	0.65
≥1.1 mmol/L (n_e=23)	1.00	0.42 (0.17—1.01)	0.08 (0.02—0.39)	0.41 (0.25—0.68)	0.001

Cox proportional-hazards regression analyses were performed to assess the association of plasma vitamin C concentration with risk of graft failure (n_{events}=75). Associations are shown with plasma vitamin C concentration as a continuous variable and according to tertiles of the vitamin C distribution (tertile 1, *n*=197: <35 µmol/L; tertile 2, *n*=202: 35–51 µmol/L; tertile 3, *n*=199: ≥51 µmol/L), with adjustment for age, sex, body mass index, donor age, donor sex, eGFR and proteinuria.
